# Advice-giving skills in pre-registration physiotherapy training

**DOI:** 10.1080/09593985.2023.2247485

**Published:** 2023-09-05

**Authors:** Lisa Osborn-Jenkins, Elizabeth Day, Hayley Payne, Robin White, Lisa Roberts

**Affiliations:** a Therapy Services, Department, University Hospital Southampton NHS Foundation Trust, Hampshire, UK; b School of Health Sciences, University of Southampton, Hampshire, UK

**Keywords:** Qualitative, physiotherapy training, advice, communication, self-management

## Abstract

**Background:**

With increased emphasis on self-management in healthcare, clinicians need outstanding skills in offering advice and empowering patients to attain an optimal outcome.

**Objectives:**

This study explores how undergraduate physiotherapists acquire knowledge, skills, and confidence to offer advice to patients in clinical practice.

**Methods:**

Convenience sampling was used to recruit 50 BSc and MSc pre-registration physiotherapy students across all years of study in one university in southern England, UK. Semi-structured interviews were conducted for first year BSc students (*n* = 13). Six focus groups of mixed BSc and MSc students were conducted, three groups (*n* = 15 students) were mid-training, and three groups (*n* = 22 students) were in their final year.

**Results:**

Thematic analysis identified 6 themes: advice content; a patient-centered approach; delivery; acquisitions; perceptions; and uptake of advice. Students placed high value on advice-giving, drawing upon multiple learning opportunities, however they felt under-prepared to deliver this skill in practice. Furthermore, perceptions of their student status, and pressures to perform on graded placements were reported to influence the advice they offered to patients.

**Conclusions:**

Developing high-level skills in promoting self-management is essential in physiotherapy, this study highlights the challenges for students to develop these skills. Academic and practice educators must explicitly enable and support students to develop the knowledge and skills to confidently offer high-quality advice to patients.

## Introduction

Encouraging people to engage in self-management and be active is one of the priorities set out in the National Health Service (2019) Long-Term Plan, and every healthcare contact is an opportunity to positively influence a patient’s health. People with long-term conditions account for “about 50% of all general practitioner appointments, 64% of all outpatient appointments and over 70% of all inpatient bed days” (Department of Health and Social Care, 2012). A key component in managing long-term conditions in routine care is to support people to self-manage as outlined in the “Making Every Contact Count” initiative (Health Education England, 2016) and clinical guideline (National Institute for Health and Care Excellence, 2014). These brief interventions include topics such as stopping smoking, improving diet, increasing physical activity, losing weight and reducing alcohol consumption to promote healthy lifestyle changes. The Healthy Conversations Project surveyed 1016 qualified allied health professionals regarding their role in public health advice, and 87.6% of “over 2000” respondents agreed that their role should include an element of preventing ill health, while 76% agreed their role provided this opportunity (Royal Society for Public Health, 2015). With first-contact roles in primary care, physiotherapists are ideally placed to deliver tailored self-management advice (National Health Service, 2017) through healthy conversations.

Despite encouragement to engage in healthy conversations (Bancroft and Moss, 2016; National Health Service, 2016) no consensus has been reported on how physiotherapists promote healthy lifestyles (Killingback et al., 2022; Shore and Hebron, 2020). Approaches used by physiotherapists to deliver advice have been shown to be inconsistent, conflicting and have not addressed long-term health promotion (Lowe, Littlewood, and McLean, 2018; Osborn-Jenkins and Roberts, 2021). Opportunities to promote self-management and healthy behaviors were missed in an observational study exploring advice offered by physiotherapists to 25 people with LBP in primary care (Osborn-Jenkins and Roberts, 2021). Moreover, physiotherapists working in in-patient settings described low confidence in delivering health promotion (citing time and discharge pressures) to deliver brief interventions effectively (Walkeden and Walker, 2015). Meanwhile outpatient musculoskeletal physiotherapists specifically reported a lack of confidence in non-exercise-based healthy conversations (Shore and Hebron, 2020). Thus, the reasons for deficiencies in delivering advice appear to be complex and multi-factorial.

According to the Oxford English Dictionary (2023), advice is a broader term to describe ”the act of offering an opinion on what recommended action to take”. Advice giving is considered a form of supportive communication (Goldsmith and Fitch, 1997) characterized by persuasive informational support (Rafferty and Beck, 2020; Thompson and O’Hair, 2008). In healthcare consultations self-management advice may include addressing general health and well-being behaviors in addition to education around condition specific actions or recommendations. The extent to which patients consider clinicians behavior change advice as acceptable is determined by the way in which the advice is framed as a treatment plan for their condition (Bergen, 2020). Effecting behavioral change is an active, iterative process, and patient education is shifting from the traditional paternalistic transfer of knowledge to a patient-centered or “people-focused” partnership. This process uses the biopsychosocial model to actively listen to patients about their concerns and fears, while incorporating skills in managing medicines, behaviors, roles, and emotions, addressing health literacy, with simple language that patients can understand (Wittink and Oosterhaven, 2018). In their discourse analysis observing 31 natural patient-nurse interactions within an acute hospital setting Crawford, Roger, and Canalin (2018) suggested that information-giving alone is insufficient to evoke change. Their ethnographically-orientated analysis highlighted the importance of communication skills; patient preferences; clinically reasoning; and patient education, in supporting patients’ learning, and optimizing outcome (Crawford, Roger, and Canalin, 2018). Patient education implies offering condition-specific information (Hutting, Oswald, Staal, and Heerkens, 2020), whereas self-management is considered a dynamic process comprising four components: i) establishing meaningful connections for patient-centered care; ii) shared-decision-making; iii) self-management support; and iv) patient-centered communication (Hutting et al., 2022). Whilst patient education remains dominant in physiotherapy consultations, self-management skills are rarely well-addressed, and so research to develop tools to assess patients’ self-management skills has been recommended (Hutting, Oswald, Staal, and Heerkens, 2020). With increased awareness of the importance of self-management, clinicians must prioritize behavioral change as an essential component of care (Lewis, Ridehalgh, Moore, and Hall, 2021; Solvang and Fougner, 2016) using coaching and communication skills to empower patients to self-manage (Hutting, Johnston, Staal, and Heerkens, 2019; Lewis, Stokes, Gojanovic, Gellatly, Mbada, Sharma, Diener and O'Sullivan 2021).

This paradigm shift needs to be prioritized in undergraduate physiotherapy training, to prepare students for the workplace, and perceived gaps in the curriculum have been identified. For example, in a single-site study of 19 final year allied health students (including 9 physiotherapists), participants reported a lack of confidence to provide appropriate public health advice (McLean, Charlesworth, May, and Pollard, 2018). Students highlighted the lack of opportunities to engage in difficult conversations with patients during clinical placements, as a limitation of their training, and expressed concerns about potentially jeopardizing the relationship with their patients, especially when addressing smoking cessation and obesity. This study also highlights the need for training in public health and healthy conversations within the academic curriculum, evidenced by McMahon and Connolly (2013), who concluded from their survey of 526 physiotherapists in Ireland, that pre and post-registration education should be reviewed to ensure physiotherapists have the knowledge-base and skills required to successfully deliver health promotion advice.

In summary, there is a current mismatch between the growing emphasis on self-management within healthcare, and the development of physiotherapists in training to deliver high-quality self-management advice with confidence. Therefore, the aim of this study was to identify how undergraduate physiotherapists acquire knowledge, skills, and confidence to offer advice for patients to inform curricula and placement development.

## Methods

### Study design

This research comprised three discrete, concurrent cross-sectional qualitative studies, sampling students in multiple cohorts, at different stages of their training, to answer the research question: “*How do student physiotherapists learn and develop knowledge, skills and confidence when giving advice to patients in clinical practice?”.* A phenomenological approach was employed to describe student physiotherapists’ clinical experiences and sense-making (Braun and Clarke, 2006) and the cross-sectional study design was used to explore differences and similarities between physiotherapy students at different stages of training (Hall, 2008). The physiotherapy program is delivered via two parallel routes (i.e. BSc and pre-registration MSc). While the academic content is delivered and assessed at different levels (as students in the MSc route already have a relevant Bachelor's degree), the clinical placements are not.

### Study 1: Early stage of training - year 1 Bsc physiotherapy students (ED)

This study comprised semi-structured interviews with students in their first term of the 3-year BSc physiotherapy program to explore the experiences they draw on prior to training and clinical placements. Given the variation in life experience among students and the early stage in training (meaning students had limited opportunity to build trusting relationships with peers and feel comfortable sharing personal experiences), interviews were chosen in preference to focus groups so that students could discuss their opinions openly (Mansell et al., 2004; Morgan, 1998). Semi-structured interviews allowed for a more flexible approach to explore first year students’ experiences, opinions and emerging themes and permitted them to express themselves freely (Boeije, 2010) and allowed for discovery of new issues not anticipated by the interviewer (Braun and Clarke, 2013).

### Study 2: Mid-stage of training - year 2 Bsc physiotherapy students and year 1 pre-registration MSc physiotherapy student (HP)

This study comprised three focus groups of students, mid-way through training (i.e. students had undertaken between 1 and 3 clinical placements), to explore their experiences and thoughts about advice-giving using the group dynamics (Freeman, 2006).

### Study 3: Final year training - year 3 Bsc physiotherapy students and year 2 pre-registration MSc physiotherapy students (RW)

Final year students, who had completed most of their required 1000 clinical hours for registration (up to 5 clinical placements), participated in one of the three focus groups. At this stage of training, students from both courses could draw upon their clinical experiences and share them in the focus groups.

### Participant recruitment

Convenience sampling was used to recruit participants for all three studies. All students across the physiotherapy programs (*N* = 128) were approached by the researchers via a spoken advert in lectures. Ethical approval was granted by the University of Southampton (Reference # ERGO52496) and to minimize coercion all students were explicitly informed that their participation was voluntary and that they had the right to withdraw from the research at any time without giving a reason and without prejudice. Participants registered their interest via a sign-up sheet to avoid unsolicited e-mails and were then emailed the participant information sheet, consent form and a choice of data collection times. The inclusion criteria for the study ensured that the student was: enrolled in the University’s physiotherapy degree during the 2019/2020 academic year; aged ≥18 yrs; able to independently communicate in English; and provide informed consent. Student members of the research team were excluded from participating in the final year focus groups, as they facilitated the groups (Table 1).

**Table 1.** Research team involvement in data collection and analysis.

Researcher	Protocol	Pilot	Study 1 interviews	Study 2 interviews	Study 3 interviews	Initial analysis	Combine analysis
ED - Year 3 BSc physiotherapy student	✓	✓	✓			✓	✓
HP - Year 3 BSc physiotherapy student	✓	✓		✓		✓	✓
RW - Year 3 BSc physiotherapy student	✓	✓			✓	✓	✓
LOJ - Senior physiotherapist and early career researcher	✓					✓	✓
LR – Consultant physiotherapist and senior researcher	✓	✓				✓	✓

### Data collection

The interview schedule and focus group topic guides (Appendix) were developed by the research team. To gain face and content validity 5 senior physiotherapists were invited to review the topic guides as they had experience in assessing students’ performance on clinical placements and could thus provide feedback on the clinical relevance of the proposed questions. Three pilot studies were undertaken, one for each study with a total of 14 allied health professional students (i.e. *n* = 2 interviewed and *n* = 12 in focus groups of mixed occupational therapy and podiatry students) at a comparable stage of training, to preserve the physiotherapy cohort for the main study. Feedback from the pilot studies informed minor changes in process and provided the novice interviewers with an opportunity to experience running a focus group prior to data collection and to develop confidence in their interviewing technique (Holloway, 2008). The novice interviewers were supported by the senior researcher (LR) and received bespoke training and feedback following the pilot studies. Piloting also ensured the topic guides had face and content validity, assessing what they intended to, and that all the desired areas were addressed (Streiner, Norman, and Cairney, 2015).

The interviews and focus groups were conducted onsite at the university between October 2019 and January 2020 and with express consent from participants were audio-recorded and transcribed verbatim. Participants in studies 2 and 3 were offered a choice of times to attend, which was a pragmatic decision that aimed to maximize participation. Care was taken to ensure no identifiable data were recorded and identification numbers were assigned to participants to further minimize any identifiable features. Each focus group was observed by another member of the research group, to provide contextual field notes, to aid transcription, reflexivity, and analyses (Table 1). As novice researchers, working on this topic as a group (albeit each with their own participants) increased their exposure to data collection and analysis.

### Data analysis

**Figure 1.**
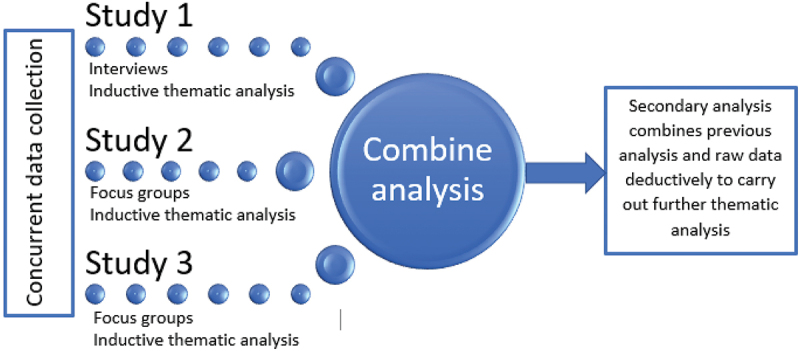
Thematic analysis process combining 3 study cohorts.

Transcribed data were thematically analyzed, using Braun and Clarke (2006) six steps: 1) familiarization with the data; 2) initial coding; 3) searching for themes; 4) reviewing themes; 5) defining themes; and 6) writing the report, and the data were managed using a Framework matrix (Gale et al., 2013). The transcripts were initially inductively analyzed by the researcher who had collected the data and senior research team (LR and LOJ) to develop new themes and insights for each study. The three studies were initially analyzed separately to ensure an inductive approach to each cohort, as data collection and analysis were conducted concurrently by the study interviewers (Figure 1). Only when this stage was complete did further analysis of the whole corpus of data occur in a central framework, in a Microsoft Excel spreadsheet, led by the first author (LOJ). The COREQ standardized reporting guidelines (Tong, Sainsbury, and Craig, 2007) were followed in both the conduct and reporting of this research, to aid transparency and rigor. No claims were made about data saturation, due to the unique experiences of clinical placements.

## Results

### Participants

The total data set for the combined 3 studies comprised 50 students. This included 13 interviews (mean duration 35 minutes, range 20–50 minutes) and 6 focus groups (mean duration 44 minutes, range 30–60 minutes) (Table 2).

**Table 2.** Data collection methods and demographic details of participants in each study

	STUDY 1 - (S1)	STUDY 2 - (S2)	STUDY 3 - (S3)
Data collection method	Interviews	3 focus groups	3 focus groups
Stage of training	Early	Mid	Final year
Participants number	13 BSc students	15 BSc students	22 BSc students
		S2 focus group participants: S2 1) 2 MSc, 3 BSc 2) 5 MSc, 2 BSc 3) 3 BSc	S3 focus group participants: S3 1) 1 MSc, 6 BSc 2) 7 BSc 3) 3 MSc, 5 BSc
Gender (male: female)	4:9	S2 total = 9:6 S2.1 = 3:2 S2.2 = 4:3 S2.3 = 2:1	S3 total = 8:10 S3.1 = 2:5 S3.2 = 2:5 S3.3 = 4:4
Length of interview	Range 20-50 minutes per interview.	Range 35-59 minutes	Range 30-60 minutes
Total duration of Audio-Data)	452	141	124

S2= study 2 focus groups (first focus group S2.1, second focus group S2.2, third focus group S2.3); S3= study 3 focus groups (first focus group S3.1, second focus group S3.2, third focus group S3.3)

### Findings

Data collection resulted in rich discussion about advice-giving in physiotherapy practice. The key topics identified were consistent throughout the three studies, however the reported impact varied, depending on the participants’ stage of training. First year students spoke broadly about the specific content of advice, the process and format of delivery, with an insight into the issues surrounding adherence and barriers to delivering advice. Learning opportunities were discussed by these students who were all at an early stage of training, with an awareness of the personal attributes required for advice-giving. Students at the mid-stage of training focused more on experiences during placements, exposure to advice-giving, general life experience and pre-university experience. They discussed the role of evidence-based practice with an awareness of different sources of evidence and varying experiences that inform advice-giving. Meanwhile, final year students strongly focused on the challenges of their status as a student on placement. The pressures of being assessed on placement was reported by student to impact (both positively and negatively) their confidence to offer advice to patients in clinical practice, especially when actioned in front of their practice educator. From the data across the 3 studies, 6 main themes were identified, summarized in Table 3. The findings reported are supported by direct quotes which are referenced according to the: stage of training; interview (I) or focus group (FG) number (1–13); and participant ID.

**Table 3.** Table of themes and sub-themes.

Theme 1: Content of advice	Theme 2: Patient-centered	Theme 3: Delivery of advice	Theme 4: Acquisitions	Theme 5: Perceptions	Theme 6: Uptake of advice
1.1 signposting 1.2 wellbeing and lifestyle *1.2.1 exercise* *1.2.2 mental health* 1.3 education and addressing previous beliefs 1.4 safety	2.1 understanding patients’ needs 2.2 tailoring advice to the patient 2.3 patient characteristics 2.4 collaboration	3.1 method of delivery 3.2 communication style 3.3 salesmanship 3.4 power of words 3.5 being honest	4.1 experience 4.2 learning resources 4.3 knowledge 4.4 confidence	5.1 perceptions of being a student 5.2 perceptions of patients 5.3 perceptions of educator/supervisor 5.4 perceptions of advice importance as modality	6.1 adherence 6.2 understanding 6.3 facilitators 6.4 barriers

Key findings from each of the studies are presented. No attempt was made to standardize the number of quotes in each theme, as the selection depended on the data in each theme. Where deviant cases were identified, additional quotes were included to highlight the range of views expressed.

### Theme 1: Content of advice

Students described the content and delivery of the advice they had either seen being given by others in clinical practice or that they had delivered during their placements such as this signposting of services.

I’ve seen leaflets [sic] been given out for the Red Cross and befriending services and stuff and also the ones about the pendent alarms (mid-training FG2:P2).

Students also identified that advice can be related to safety such as “preventing falls”, and what to do about “red flag symptoms”. Students recognized different types of well-being advice.

… a good level of baseline advice that we all give regardless like exercises, nutrition and mental well-being (mid-training FG2:P4).

Students recognized that the content of advice may be specific or generic in nature.

Advice on smoking and recreational drugs because you ask how many they smoke a day … so sort of health and lifestyle things (mid-training FG1:P5).

It was suggested that physical and mental health are both important areas of clinical practice where physiotherapists offer advice. Students discussed the benefits and consequences, sharing examples of different advice content and signposting, although when explicitly mentioned, there was uncertainty as to whether education was considered part of advice.

… I suppose that’s more sort of educating, I’m not sure if that sort of falls under advice, I don’t know [watching an advice video about condition management] (early-training I:P2).

### Theme 2: Patient-centered

Students highlighted the importance of “understanding the patient” to provide a foundation on which to formulate the advice. This was achieved by focusing their listening and respecting the patient as a unique individual with bespoke needs.

… part of it is listening to that person … understanding, what they want to achieve, um and their hobbies and interests…and relating it to that … working together to achieve goals (early-training I:P11).

Furthermore, “tailoring” advice was considered a process to ensure advice offered was specific and relevant, adapted to the patients’ needs and preferences, including work, home-life, hobbies and goals. The concept of patient choice, when offering advice as a treatment modality, was explored by the final year students, showing an awareness that not only the content may need tailoring, but the scope and quantity of the advice too.

[It’s] important to be adaptable … not every patient will tolerate the same amount of advice giving. Some may want to know more information, some might not even really want to know any of it at all (final-year FG1:P5).

Students observed that tailoring advice to the patient rather than their condition was key to improving uptake. They recognize each patient as an individual, and therefore partnering with the patient is key to optimize the chance of behavior change.

[Supervisors are] good at getting patients to adhere to some advice because they’ve tailored it! (final-year FG1:P2).

What could work for one person … may not work for another... being able to understand that is a big thing (early-training I:P2).

### Theme 3: Delivery of advice

The delivery of advice was seen as a separate skill and discussed in all 3 studies. Communication was described as “a vital tool”, with importance placed on having adaptive skills and being able to “communicate well”. Students described a spectrum of approaches to communicate and deliver advice, ranging from “providing a stern-talking to” and “being firm”, to “being non-confrontational”, depending on the patients’ characteristics.

I say it to people with aggression, but more like tough love … (mid-training F3:P3).

… I’m never going to have a professional authoritative voice talking to people really loudly… I hate that (mid-training F3:P2).

A confident delivery was favored by final year students and was described as a form of “salesmanship” to create buy-in with patients. However, this felt uncomfortable for some:

It’s almost a form of salesmanship, if you don’t seem confident in saying it yourself, the patient will pick that up (final-year FG1:P1).

It’s so artificial, it’s so hard to erm like give advice properly because it’s almost cringeworthy (final-year FG1:P2).

Furthermore, students were aware of the power of words and raised concerns about saying “the wrong thing”. They reported this not only affected the delivery but recognized the likelihood that their choice of wording might affect patients’ future beliefs and attitudes.

How one singular word can have like a catastrophic effect … wear and repair instead of wear and tear (final-year FG1:P2).

These situations generated discussion about the importance of being honest with patients, as students acknowledged they cannot know everything. They recognized honesty to be highly-valued by patients along with building trust and rapport.

[I say] I might have to go away and … look up something else, they appreciate that honesty (final-year FG1:P5).

### Theme 4: Acquisitions

Students identified a range of learning opportunities and ways to gain experience in advice-giving and were able to identify gaps in their own knowledge. They recognized the need to be able to impart information with confidence, and accepted these skills develop by investing time and practice, gaining further experience. Physical resources were identified more readily as opportunities for gaining knowledge, than the personal processes of learning which included reflecting on their own experience and exposure time. Among the predominantly digital resources were apps, podcasts, online videos, and social media sites, which enabled access to content and role models to emulate.

the internet, that’s where I get most of my stuff (final-year FG1:P3).

a lot of social media and stuff … scrolling through Instagram … some of them kind of explain things really well (early-training I:P9).

Moreover, final year students suggested other methods for information gathering, including formal observation and asking others.

I made sure that I went and like shadowed a few of them in the team (final-year FG2:P7).

I guess asking … either a friend or supervisor (final-year FG3:P8).

Evidence-based, reliable resources, such as NICE guidelines, were highlighted by students who were midway through their training. In contrast, first year students discussed sourcing “Google” as an alternative to asking someone in a clinical setting, for its immediacy and potential in reducing the students’ need to retain information. The interval between the learning opportunity and chance to practice the skills appears to be important to students, and recent acquisition of knowledge was considered to aid delivery.

If you spend your weekend doing a knee course … on the Monday morning if you had a knee patient, great! you are pretty comfortable with the advice that you would give (final-year FG2:P3).

Students preferred learning opportunities in clinical practice to university-based learning that included simulation and role-play and suggested that feedback from patients helped to consolidate knowledge and improve their confidence.

[role play] gave me a chance to make errors and I’d have to correct things I was saying, so when I did it again, it was like the second go as opposed to it being my first time (final-year [FG1:P5).

… if you’ve given someone advice and they come back to you and they say oh I did that and it worked really well, that kind of helps boost your confidence and self-efficacy going forwards (mid-training FG1: P2).

Student identified the importance of previous life experiences in preparing their ability to offer advice effectively, drawing from personal experiences

It [confidence to offer advice] comes from life experience and can’t be taught to you … [its] just a matter of experience, and a matter of time, on how to give good advice (final-year FG2:P6).

[good advice is] based on past experiences (final-year FG3:P3).

I came straight from A-levels, I never had that sort of experience and questioning someone about behavior changes and things. It just seemed really unnatural to me (mid-training F3: P2).

I’ve had counseling sessions … their tone and how they speak … actually being in kind of the patient’s point of view [acquiring skills] (mid-training F2:P1).

First year students attributed their skills acquisition to being in a clinical environment and observing practice as a main source of learning, although they seemed unclear how this learning would occur.

I suppose those skills just naturally develop when you’re actually out on placement in your own practice (early-training I:P4).

### Theme 5: Perceptions

Students through all stages of training perceived their status as a physiotherapy student may influence their ability to offer advice to patients effectively, in terms of how their patients and supervisors perceived them, and also their desire to appear confident and gain trust. This perception was repeated by many students throughout the program.

… If you’re not confident when you’re giving advice … then no-one’s going to believe you (early-training I:P10).

[Supervisors] doubting your knowledge in front of your patient is the most … soul-destroying thing ever because … you just [lost] their respect (final-year FG2:P6).

It was concerning that students reported that appearing confident was more important than the content of the advice:

… be confident with what you’re saying, so even if what you’re saying is complete rubbish, they believe you (early-training I:P13).

It was important to students how their patients viewed them, and they linked this to patients’ past experience of being treated by other students, resulting in the students perceiving additional pressure:

If they don’t have a very good opinion of being treated by a student, and they’re just not very open to what you’re saying, and they’re switched off and not engaging, then that immediately drops my confidence levels (mid-training FG1:P3).

Students cite needing external positive reinforcement as a key factor in building their confidence:

the way I get confidence is through like positive reinforcement, someone’s gotta tell me I’m doing the right thing (early-training I:P5).

Furthermore, experienced students highlighted the importance of first impressions. They felt even being introduced as a “student” affected their own confidence and the professional relationship with patients, with patient losing trust in the students’ abilities. Preference was shown in being introduced as part of the team, to increase engagement and to strengthen their relationship.

When I get introduced as a member of the physio team immediately, they have more engagement and like more faith … [than] if I was introduced as a student (final-year FG2:P6).

Your patient knows that you’re a student … they almost don’t believe you, so then you almost don’t believe yourself (final-year FG1:P3).

Additionally, final year students reflected on the role the practice educator had when they were also present in the consultation. This resulted in students feeling inadequate and further reduced their confidence.

Know someone is watching you and [although] you know what to do … you are just constantly aware of that (final-year FG1:P1).

Realistically all they [patients] want to hear is something that comes out of [your] supervisors’ mouth. So you … feel, like, insufficient (final-year FG1:P2).

With the sustained pressure of their own performance being assessed, students admitted to tailoring advice to their supervisors rather than patients, as great importance is placed on pleasing the person marking them, even if students did not agree with the advice. Practice educators presence appears to have a strong influence on the advice students offered to patients:

[It] feels like its scripted to what your supervisor wants you to say … because they are the ones assessing you (final-year FG1:P1).

… you’re there to learn, but you’re [also] there to get graded by them … so [you] give advice that you might not agree with (final-year FG2:P4).

Students who are assessment-focused may further limit their learning experience on placement in fear of getting things wrong.

I think it depends who I am with as well. I think if I’m with my supervisor, I wouldn’t feel as confident at giving advice because I’m worried it’s going to be wrong (mid-training FG2:P7).

The fear of “getting it wrong” went beyond grades and more to concerns of what would happen if students gave the wrong advice to the patient. They craved reassurance and affirmation that the advice they offered was appropriate and accurate:

No matter how confident I’d be like, if I give the wrong advice and something else happened to that person … (final-year FG2:P2).

and I suppose if it’s NICE (guidelines), that you can pretty much be sure that your allowed to say that and that’s ok to kind of give that advice so (mid-training F1:P4).

The concern students expressed about giving “the wrong advice” highlighted the high-regard students placed on advice-giving in practice. They considered it to be of the utmost importance, as a key skill of physiotherapists:

I think [advice is] part of a huge drive toward increasing self-efficacy and self-management (final-year FG3:P3).

I think [advice is] at the top to be honest because … [it] can change their whole life (final-year FG1:P6).

### Theme 6: Uptake of advice

Despite students acknowledging the importance of empowering patients to self-manage, several barriers were identified in the uptake of advice offered including: extrinsic factors (such as a lack of patient engagement, language differences, and patients not feeling empowered to say they do not understand):

… if someone says to you do you understand some people just say yes … it’s just easier than just saying I don’t understand (mid-training F1: P5).

In contrast, students identified intrinsic challenges such as their difficulties discussing sensitive issues with patients and feeling unqualified to advise, based on their own life experience. Difficult topics included death, cultural differences and public health advice. Students recognized they may struggle to empathize with a patient and offer some health advice, due to differences in life experiences and some considered their young age to be a barrier to patients taking on board their advice.

I had to give advice to someone about giving up smoking, which I found really difficult having never smoked. I couldn’t, I can’t relate how difficult it is to stop smoking (final-year FG1:P4).

We’ve not been mums … I can’t even begin to understand that because I don’t have a child (final-year FG2:P2).

A confident delivery of offering advice to promote understanding and empower patients was reported by students to facilitate adherence and self-efficacy.

if you’re … not showing confidence, they’re likely not to trust you (early-training I:7).

It’s about empowering the patient isn’t it, for self-management … lectures on that help... I guess the biggest thing … to get them to do that.” (mid-taining F2:P5).

[advice is] … ”huge drive toward increasing self-efficacy and self-management in patients because they [patients] need to have the understanding on why they do things, what they are doing … to help (final-year F3:P3).

## Discussion

Physiotherapy students recognized the importance of advice-giving to empower patients to self-manage and identified a range of advice-giving experiences and ways in which these skills are developed. While the focus of topics differed between students at various stages of training they shared a common feeling of being under-prepared in offering advice and in keeping with the literature (McLean, Charlesworth, May, and Pollard, 2018; McMahon et al., 2016). Students reported a lack of under-pinning theoretical teaching on public health advice in their academic programs to help them feel prepared. While the curriculum includes activities such as role-play to help students develop their skills in a safe environment and learn from mistakes. They still lacked confidence in offering advice, despite previous life/work experience (and/or volunteering) that may be considered helpful in mitigating for this. Like McMahon et al. (2016) physiotherapy students preferred learning opportunities during clinical placements, rather than the academic program, to gain knowledge and confidence, citing feedback from patients as a key learning requirement. Mistakes were seen by some as an opportunity for learning and development, which concurred with Ferretti, Rohde, Moore, and Daboval (2019) who reported this to be crucial in professional learning opportunities. Furthermore, in keeping with Stainsby and Bannigan (2012), students considered lone working increased their confidence, as they did not feel so pressured about “getting it wrong” and learning from mistakes, as these were unlikely to be detected or influence their placement grade.

Online resources also helped students gain knowledge on advice topics, and various formats were described, including official electronic documents, podcasts and social media, in addition to Google searches. The way in which health professionals consume informal professional education is changing, with increasing usage of electronic formats and variability in whether physiotherapists critically appraise the information accessed (Clode, Darlow, Rouse, and Perry, 2021). It is unclear in the literature how student physiotherapists consume digital education or how they appraise this knowledge or emulate prominent digital figures/role models. Despite social media being a possible effective educational medium (Maloney et al., 2015), some students were wary of reporting bias and quality. They considered guidelines to be evidence-based knowledge that they were “allowed to say,” and worried that saying the wrong thing may have repercussions that could result in litigation, or harm to the patient.

**Figure 2.**
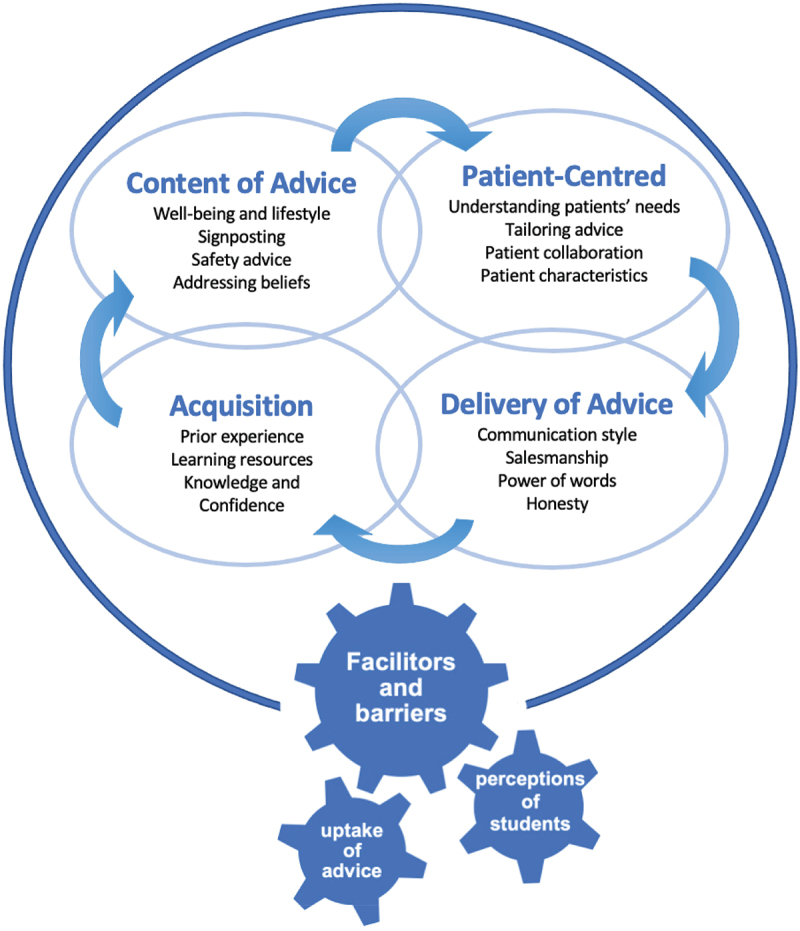
Factors influencing advice-giving skills in pre-registration physiotherapy students.

Learning to offer advice in clinical practice includes both the knowledge of advice content and learning about communication skills in delivering this in an individualized approach to patients. There appears to be a cyclical nature to the factors influencing advice-giving skills in physiotherapy training (Figure 2) whereby the acquisition of skills enables student physiotherapists to explore the content of advice and tailor it in a patient-centered way. The delivery of advice was seen as a separate and “vital tool” with varying approaches to communicating advice deemed necessary to encourage uptake and behavior change. Students placed high-value in a confident delivery of messages such as “salesmanship” or “tough love”. Rentmeester, Brack, and Kavan (2007) suggested that in healthcare, “tough love” is a way in which professional caregivers’ express duty of care and if done skillfully, can be beneficial. This was in contrast to findings from reported strategies of advice delivery including cognitive reassurance to reassure, encourage, explain and prepare patients when delivering advice (Osborn-Jenkins and Roberts, 2021). Therefore “perceptions” and “uptake of advice” may both act as facilitators or barriers to offering advice and the cyclical nature of developing advice-giving skills (Figure 2).

The uptake of advice was reported to be influenced both positively and negatively by extrinsic factors, such as patients’ engagement and understanding of the advice offered. Meanwhile the students’ perceptions of their own status, the importance of the advice, their practice educator, and the patient all positively and negatively impacted upon the advice given.

Positive feedback from patients and supervisors boosted students’ confidence and self-efficacy in offering advice. Physiotherapy students described “soul-destroying” experiences when supervisors doubted their knowledge and made them feel inadequate in front of patients. They reported tailoring their advice to their supervisor more than the patient as they needed to pass the placement. This contradicted views expressed by some students who feared giving the “wrong advice” and the impact this may have on patients. Conflicting views were also expressed with high value placed on honesty in advice-giving and also the need to deliver a confident message, even if they lacked confidence in the correctness of the content. Students particularly struggled when sign-posting patients to other sources of help or when discussing public health advice, especially for topics outside their own life experiences, and feared their student status may have a negative impact.

### Implications for policy and practice

As the population presents with increasingly complex health issues, the need to be able to confidently offer and deliver advice to patients has never been greater. This study has identified the need for knowledge and communication skills in academic physiotherapy programs, and a need for greater focus on advice-giving in clinical placements, as clinicians work with students to ensure they have the knowledge, skills and confidence to offer advice to patients and enable them to self-manage. Further research is required from a pedagogical perspective to understand how to support students learning to offer advice. It is also essential that these skills are learned alongside strategies to promote behavior change in patient, to optimize self-management strategies in clinical practice.

### Study strengths and limitations

While this qualitative study includes the views of pre-registration physiotherapists at all stages of training, and makes important contributions to the evidence base, it does have several limitations. Firstly, it was a single-site study and, while the authors included 50 students from both the BSc and pre-registration MSc programs, students do have diverse clinical placements in varied geographic locations. This mixed cohort have diverse prior life experience, but further demographic information was not recorded. However, the findings should be generalized with caution, as they were derived from students in one university in the UK, therefore limiting variation in academic preparations for placement and prior life experience an unknown factor. Despite this study being UK-based research, there remains an international need for more evidence in the field.

Secondly, given the radical changes to education formats and the rapid implementation of virtual and simulation training in response to the pandemic in 2020 (Tognon, Grudzinskas, and Chipchase, 2021; World Physiotherapy, 2020) the data were collected pre-pandemic and therefore changes to the course format and delivery may change views. Simulation is not a new concept in physiotherapy education, offering bespoke patient scenarios, guaranteed wide exposure and real-time feedback for corrective learning, and improving interprofessional teamwork (Blackstock and Jull, 2007; Dennis, Furness, Duggan, and Critchett, 2017). Simulation may even enable academic courses to provide students the necessary opportunities to learn through reflection and real-time feedback, described as helpful in this study. This form of learning by immersive simulation has been shown to enable students to transition to work in clinical environments. However, a study conducted in Australia showed that although clinical competency significantly improved, students’ self-reported confidence (including communications skills) was not translated and developed across new clinical areas (Wright et al., 2018). In contrast, simulation learning, which through necessity has been rapidly implemented, is reported to have transitioned well over remote delivery (Evans, Douglas, Moffatt, and Harvey-Dunstan, 2020) but must focus on translating communication skills across all areas of physiotherapy practice.

## Conclusion

Physiotherapy students primarily learn about offering advice to patients through their clinical placements but identified a lack of under-pinning theoretical teaching on public health advice in their academic programs. Furthermore, this research highlights a need for greater emphasis on communication skills to help students prepare for placements and enhance their confidence when offering advice to patients in clinical practice.

## Appendix

**Table:**

Study 1. Interview Schedule
1. What do you think advice giving is in clinical practice? 2. Can you describe the types of advice that you’ve seen physiotherapists offer patients in a clinical setting? 3. How are you learning to develop these skills? 4. Do you have any experiences from before you started your physiotherapy training that you think will help you develop these skills? 5. Are there any resources you have used to help increase your knowledge in advice giving? 6. How confident do you feel giving advice to patients? 7. Do you think that you’ve got any particular strengths or weaknesses in giving advice if so what are they? 8. Can you give me an example of an experience of advice giving that you think went well? 9. Can you give me an example of an experience of advice giving that you think didn’t go particularly well? 10. Compared to other treatments that physiotherapists use (i.e. exercise prescription, manual therapy, gait reeducation, mobilizing, and electrotherapy) where do you think advice ranks, in terms of its importance? 11. What fears do you have about giving advice? 12. Is there anything else that we haven’t talked about that you feel is important in relation to developing knowledge, skills and confidence when offering advice to patients?

**Table:**

Study 2 and 3 Focus group Interview Schedule
**1. How do you feel about giving advice?** [*Probes: Can you tell me a little bit more about … ? Is there anything else you would like to add? You mentioned … could you tell me a little bit more about … ?]* **2. Can you describe the types of advice that you’ve seen physiotherapists offer patients in a clinical setting – this can be while you were on work experience or clinical placements?** *[Probes: Is there anything else? Advice to keep active, increase exercise/prevention of re-injury advice/public health and lifestyle advice – smoking cessation, healthy eating/top tips for managing activities of daily living/improving posture/where to find information and support groups]* **3. Can you tell me about the types of advice you’ve offered to patients while you were on a clinical placement?** *[Probes: Is there anything else? Advice to keep active, increase exercise/prevention of re-injury advice/public health and lifestyle advice – smoking cessation, healthy eating/top tips for managing activities of daily living/improving posture/where to find support groups.]* **4. How are you learning to develop these skills?** *[Probes: Where have you learned this (university modules/placements/previous experience/external courses or other opportunities?]* **5. Are there any resources you have used to help increase your knowledge in advice giving?** *[Probes: Is there a particular person who has influenced you? Any other resources (videos/feedback/role-play that you found particularly helpful)? Going forwards how do you feel you could further increase your knowledge in this area?]* **6. How confident do you feel giving advice to patients?** *[Probes: What has helped you gain this confidence? How did you feel on your first placement? How easy is it to transfer these skills between different types of placements? What could be done to help improve your confidence?]* **7. Can you give me an example of an experience of advice giving that you think went well?** *[Probes: Either yourself giving advice or someone else? Why do you think it went well? What have you learned from this?]* **8. Can you give me an example of an experience of advice giving that you think didn’t go particularly well?** *[Probes: Either yourself giving advice or someone else? Why do you think it didn’t go well? What do you think could have been done differently? What have you learned from this?]* **9. Compared to other treatments that physiotherapists use (i.e. exercise prescription, manual therapy, gait reeducation, mobilizing and electrotherapy) where do you think advice ranks, in terms of its importance?** **10. What fears do you have around giving advice?** [*Probes: Can you tell me a little bit more about … ? Is there anything else you would like to add? You mentioned … could you tell me a little bit more about … ?]* **1. Is there anything else that we haven’t talked about that you feel is important in relation to developing knowledge, skills and confidence when offering advice to patients?** *[General probes: Can you tell me a little bit more about … ? Is there anything else you would like to add? You mentioned … could you tell me a little bit more about … ? How did that make you feel? Can you give me any other examples of … ?]*

## References

[cit0001] Bancroft D, Moss C 2016 Making every contact count in physiotherapy: Addressing the health and wellbeing of patients, staff and the wider local community. Physiotherapy 102: e254–e255. 10.1016/j.physio.2016.10.319.

[cit0002] Bergen C 2020 The conditional legitimacy of behavior change advice in primary care. Social Science and Medicine 255: 112985. 10.1016/j.socscimed.2020.112985. 32371269

[cit0003] Blackstock F, Jull G 2007 High-fidelity patient simulation in physiotherapy education. Australian Journal of Physiotherapy 53: 3–5. 10.1016/S0004-9514(07)70056-9. 17326733

[cit0004] Boeije H 2010 Analysis in Qualitative Research. London: Sage.

[cit0005] Braun V, Clarke V 2006 Using thematic analysis in psychology. Qualitative Research in Psychology 3: 77–101. 10.1191/1478088706qp063oa.

[cit0006] Braun V, Clarke V 2013 Successful Qualitative Research a Practical Guide for Beginners. pp. 85–127. London: Sage.

[cit0007] Clode N, Darlow B, Rouse J, Perry M 2021 What electronic information resources do physiotherapists prefer to use to support their CPD. Physiotherapy Research International 26: e1881. 10.1002/pri.1881. 32964592

[cit0008] Crawford T, Roger P, Canalin S 2018 Supporting patient education using Schema-Theory: A discourse analysis. Collegian 25: 501–507. 10.1016/j.colegn.2017.12.004.

[cit0009] Dennis D, Furness A, Duggan R, Critchett S 2017 An interprofessional simulation-based learning activity for nursing and physiotherapy students. Clinical Simulation in Nursing 13: 501–510. 10.1016/j.ecns.2017.06.002.

[cit0010] Department of Health and Social Care 2012 Long-Term Conditions Compendium of Information. 3^rd^. https://www.gov.uk/government/publications/long-term-conditions-compendium-of-information-third-edition

[cit0011] Evans L, Douglas E, Moffatt F, Harvey-Dunstan T 2020 Use of remote simulation to develop undergraduate physiotherapy students’ skills in assessing the acutely ill patient. BMJ Simulation and Technology Enhanced Learning 6: A26.

[cit0012] Ferretti E, Rohde K, Moore G, Daboval T 2019 Catch the moment: The power of turning mistakes into ‘precious’ learning opportunities. Paediatrics and Child Health 24: 156–159. 10.1093/pch/pxy102. 31111832 PMC6519615

[cit0013] Freeman T 2006 Best practice in focus group research: Making sense of different views. Journal of Advanced Nursing 56: 491–497. 10.1111/j.1365-2648.2006.04043.x. 17078825

[cit0014] Gale N, Heath G, Cameron E, Rashid S, Redwood S 2013 Using the framework method for the analysis of qualitative data in multi-disciplinary health research. BMC Medical Research Methodology 13: 117. 10.1186/1471-2288-13-117. 24047204 PMC3848812

[cit0015] Goldsmith D, Fitch K 1997 The normative context of advice as social support. Human Communication Research 23: 454–476. 10.1111/j.1468-2958.1997.tb00406.x.

[cit0016] Hall J 2008 Cross-sectional survey design. In: Lavrakas P, (Ed) Encyclopedia of Survey Research Methods, pp. 173–174. Thousand Oaks, CA: Sage.

[cit0017] Health Education England 2016 Making Every Contact Count Guidance Documents. http://www.makingeverycontactcount.co.uk/evidence/guidance/.

[cit0018] Holloway I 2008 A-Z of Qualitative Research in Healthcare (2^nd^), p. 188. Chichester: Blackwell Publishing.

[cit0019] Hutting N, Caneiro JP, Ong’wen O, Miciak M, Roberts L 2022 Patient-centered care in musculoskeletal practice: Key elements to support clinicians to focus on the person. Musculoskeletal Science and Practice 57: 102434. 10.1016/j.msksp.2021.102434. 34376367

[cit0020] Hutting N, Johnston V, Staal J, Heerkens Y 2019 Promoting the use of self-management strategies for people with persistent musculoskeletal disorders: The role of physical therapists. Journal of Orthopaedic and Sports Physical Therapy 49: 212–215. 10.2519/jospt.2019.0605. 30931733

[cit0021] Hutting N, Oswald W, Staal J, Heerkens Y 2020 Self-management support for people with non-specific low back pain: A qualitative survey among physiotherapists and exercise therapists. Musculoskeletal Science and Practice 50: 102269. 10.1016/j.msksp.2020.102269. 33039797

[cit0022] Killingback C, Thompson M, Chipperfield S, Clark C, Williams J 2022 Physiotherapists’ views on their role in self-management approaches: A qualitative systematic review. Physiotherapy Theory and Practice 38: 2134–2148. 10.1080/09593985.2021.1911011. 33813990

[cit0023] Lewis J, Ridehalgh C, Moore A, Hall K 2021 This is the day your life must surely change. Physiotherapy 112: 158–162. 10.1016/j.physio.2021.05.007. 34111808

[cit0024] Lewis J, Stokes E, Gojanovic B, Gellatly P, Mbada C, Sharma S, Diener I, O’Sullivan P 2021 Reframing how we care for people with persistent non-traumatic musculoskeletal pain: Suggestions for the rehabilitation community. Physiotherapy 112: 143–149. 10.1016/j.physio.2021.04.002. 34102533

[cit0025] Lowe A, Littlewood C, McLean S 2018 Understanding physical activity promotion in physiotherapy practice: A qualitative study. Musculoskeletal Science and Practice 35: 1–7. 10.1016/j.msksp.2018.01.009. 29413948

[cit0026] Maloney S, Tunnecliff J, Morgan P, Gaida J, Clearihan L, Sadasivan S, Davies D, Ganesh S, Mohanty P, Weiner J, et al. 2015 Translating evidence into practice via social media: A mixed-methods study. Journal of Medical Internet Research 17: e242. 10.2196/jmir.4763. 26503129 PMC4642790

[cit0027] Mansell I, Bennett G, Northway R, Mead D, Moseley L 2004 The learning curve: The advantages and disadvantages in the use of focus groups as a method of data collection. Nurse Research 11: 79–88. 10.7748/nr2004.07.11.4.79.c6217. 15227901

[cit0028] McLean S, Charlesworth L, May S, Pollard N 2018 Healthcare students’ perceptions about their role, confidence and competence to deliver brief public health interventions and advice. BMC Medical Education 18: 114. 10.1186/s12909-018-1224-0. 29793485 PMC5968571

[cit0029] McMahon N, Connolly C 2013 Health promotion knowledge, attitudes and practices of chartered physiotherapists in Ireland: A national survey. Physiotherapy Practice and Research 34: 21–28. 10.3233/PPR-2012-0008.

[cit0030] McMahon S, O’Donoghue G, Doody C, O’Neill G, Barrett T, Cusack T 2016 Standing on the precipice: Evaluating final-year physiotherapy students’ perspectives of their curriculum as preparation for primary health care practice. Physiotherapy Canada 68: 188–196. 10.3138/ptc.2015-11E. 27909366 PMC5125481

[cit0031] Morgan D 1998 The Focus Group Guide Book. London: Sage Publication. 10.4135/9781483328164.

[cit0032] National Health Service 2016 Better Conversations, Better Health. http://www.betterconversation.co.uk/images/A_Better_Conversation_Resource_Guide.pdf.

[cit0033] National Health Service 2017 Allied Health Professions into Action. https://www.england.nhs.uk/wp-content/uploads/2017/01/ahp-action-transform-hlth.pdf.

[cit0034] National Health Service 2019 The NHS Long Term Plan. https://www.longtermplan.nhs.uk/publication/nhs-long-term-plan/.

[cit0035] National Institute for Health and Care Excellence 2014 Behaviour Change: Individual Approaches. https://www.nice.org.uk/guidance/ph49/resources/behaviour-change-individual-approaches-pdf-1996366337989.

[cit0036] Osborn-Jenkins L, Roberts L 2021 The advice given to physiotherapists to people with back pain in primary care. Musculoskeletal Science and Practice 55: 102403. 10.1016/j.msksp.2021.102403. 34130069

[cit0037] Oxford English Dictionary 2023 “advice, n.” OED Online, Oxford University Press, March 2023. Accessed 20 March. www.oed.com/view/Entry/2987.

[cit0038] Rafferty K, Beck G 2020 “You are not alone”: Advice giving for parents of children living with complex chronic conditions. Health Communication 35: 1386–1395. 10.1080/10410236.2019.1636341. 31264478

[cit0039] Rentmeester C, Brack A, Kavan M 2007 Third and fourth year medical students’ attitudes about and experiences with callousness: The good, the bad and the ambiguous. Medical Teacher 29: 358–364. 10.1080/01421590701418799. 17786751

[cit0040] Royal Society for Public Health 2015 Healthy Conversations and the Allied Health Professionals. https://www.rsph.org.uk/resourceLibrary/healthy-conversations-and-the-allied-health-professionals.html.

[cit0041] Shore H, Hebron C 2020 Musculoskeletal physiotherapists’ perceptions of health promotion. Musculoskeletal Science and Practice 50: 102260. 10.1016/j.msksp.2020.102260. 33010738

[cit0042] Solvang PK, Fougner M 2016 Professional roles in physiotherapy practice: Educating for self-management, relational matching, and coaching for everyday life. Physiotherapy Theory and Practice 32: 591–602. 10.1080/09593985.2016.1228018. 27710166

[cit0043] Stainsby K, Bannigan K 2012 Reviewing work-based learning opportunities in the community for physiotherapy students: An action research study. Journal of Further and Higher Education 36: 459–476. 10.1080/0309877X.2011.643769.

[cit0044] Streiner D, Norman G, Cairney J 2015 Validity. In: Health Measurement Scales: A Practical Guide to Their Development and Use (5^th^), pp. 227–253. Oxford: Oxford University Press.

[cit0045] Thompson S, O’Hair D 2008 Advice-giving and the management of uncertainty for cancer survivors. Health Communication 23: 340–348. 10.1080/10410230802229712. 18701998

[cit0046] Tognon K, Grudzinskas K, Chipchase L 2021 The assessment of clinical competence of physiotherapists during and after the COVID-19 pandemic. Journal of Physiotherapy 67: 79–81. 10.1016/j.jphys.2021.02.011. 33722499 PMC7944097

[cit0047] Tong A, Sainsbury P, Craig J 2007 Consolidated criteria for reporting qualitative research (COREQ): A 32-item checklist for interviews and focus groups. International Journal for Quality in Health Care 19: 349–357. 10.1093/intqhc/mzm042. 17872937

[cit0048] Walkeden S, Walker K 2015 Perceptions of physiotherapists about their role in health promotion at an acute hospital: A qualitative study. Physiotherapy 101: 226–231. 10.1016/j.physio.2014.06.005. 25282386

[cit0049] Wittink H, Oosterhaven J 2018 Patient education and health literacy. Musculoskeletal Science and Practice 38: 120–127. 10.1016/j.msksp.2018.06.004. 30017902

[cit0050] World Physiotherapy 2020 World Physiotherapy Response to COVID-19 May 2020 Briefing Paper 1: The Immediate Impact on the Higher Education Sector and Response to Delivering Physiotherapy Entry Level Education.https://world.physio/sites/default/files/2020-07/Education-Briefing-paper-1-HEI.pdf.

[cit0051] Wright A, Moss P, Dennis D, Harrold M, Levy S, Furness A, Reubenson A 2018 The influence of a full-time, immersive simulation-based clinical placement on physiotherapy student confidence during the transition to clinical practice. Advances in Simulation 3: 3. 10.1186/s41077-018-0062-9. 29484204 PMC5819286

